# Imatinib-induced dermatomyositis *sine dermatitis* - a rare case report

**DOI:** 10.3389/fimmu.2024.1398453

**Published:** 2024-04-29

**Authors:** Augusto Silva, Vasco C. Romão, Raquel Campanilho-Marques

**Affiliations:** ^1^ Rheumatology Department, EULAR Centre of Excellence, Unidade Local de Saúde Santa Maria, Lisbon Academic Medical Center and European Reference Network on Rare Connective Tissue and Musculoskeletal Diseases Network (ERN-ReCONNET), Lisbon, Portugal; ^2^ Instituto de Medicina Molecular João Lobo Antunes, Faculdade de Medicina da Universidade de Lisboa, Lisbon, Portugal; ^3^ Unidade de Reumatologia Pediátrica, Unidade Local de Saúde Santa Maria, Lisbon, Portugal

**Keywords:** inflammatory myopathies, dermatomyositis *sine dermatitis*, autoantibodies, proximal muscle weakness, imatinib mesylate

## Abstract

Idiopathic Inflammatory Myopathies are rare conditions with several heterogeneous disease subtypes. They can range from limited muscle or skin involvement to severe, systemic, life-threatening disease. Although the etiology is unknown, some evidence suggests a role for external agents, particularly drugs. Herein, we present a case of a 71-year-old woman with chronic myeloid leukemia who developed imatinib-induced dermatomyositis *sine dermatitis*. The presentation was predominantly muscular, characterized by proximal muscle weakness and myalgia of the lower limbs, with positive anti-Mi2a antibodies. Spontaneous recovery was observed after drug discontinuation, without the need for immunosuppressive therapy. This is the first confirmed description of an imatinib-induced dermatomyositis *sine dermatitis.* It reflects the importance of a high awareness from rheumatologists and hematologists to accurately anticipate and identify similar situations.

## 1 Introduction

Idiopathic Inflammatory Myopathies (IIM) are rare systemic inflammatory diseases, that include dermatomyositis (DM), polymyositis, immune-mediated necrotizing myopathy, overlap myositis, antisynthetase syndrome and inclusion body myositis (1–3).

DM is a clinical heterogeneous disease characterized by distinct skin lesions. These can be highly suggestive (e.g., Gottron’s papules/sign, heliotrope rash), characteristic (e.g., ragged cuticles and periungual erythema, shawl sign, V sign and holster sign), compatible (e.g., poikiloderma and periorbital edema), less common (e.g., ulcerative lesions and cutaneous vasculitis), rare (e.g., mechanic’s hands), and non-specific (Raynaud’s phenomenon). A constellation of systemic manifestations can also be present, including constitutional symptoms (weight loss, fever, night sweats) and specific organ involvement, such as pulmonary, cardiovascular, gastrointestinal or vascular involvement (4).

In patients without skin disease (DM *sine dermatitis*) or without evident myositis (amyopathic or hypomyopathic DM), DM can be difficult to diagnose (4). The European Neuromuscular Centre (ENMC) criteria recognize DM *sine dermatitis*, characterized by subacute proximal muscle weakness, marked elevation of CK and a muscle biopsy consistent with DM, while lacking cutaneous features (1, 2). Diagnosis is based on the clinical examination in combination with raised CK, presence of myositis specific/associated autoantibodies (MSA or MAA respectively), electromyography (EMG) and muscle biopsy (1). Some MSA are exclusively associated with a diagnosis of DM, namely anti-Mi2, anti-MDA5, anti-NXP2, anti-TIF1, and anti-SAE (4). The use of muscle magnetic resonance imaging (MRI) can aid in guiding muscle biopsy and in the differential diagnosis with other muscle diseases, such as muscular dystrophies (1).

The pathogenesis of DM is multifactorial, complex, and incompletely understood. Genetic, environmental, and immune mechanisms are thought to play an important role in DM development (4). The etiology remains unclear, but some external factors may be important, most notably drugs (1). Drug-induced myopathy is among the most common causes of muscle disease and includes alcohol, glucocorticoids, statins, cocaine, antimalarial drugs, antipsychotic drugs, colchicine, zidovudine, interferon, tumor necrosis factor inhibitors, and chemotherapeutic agents (e.g., gemcitabine and immune checkpoint inhibitors) (5, 6). Rare reports of inflammatory myopathy, possibly translating a hypersensitivity reaction to the drug, have been described with D-penicillamine, cimetidine, procainamide, levodopa, and phenytoin (7). Myositis has been reported only seldomly in patients with chronic myeloid leukemia (CML), and it is usually associated with hydroxyurea or alpha-interferon administration (8).

## 2 Case description

### 2.1 Demographic information and clinical findings

A 71-year-old woman, with a three-year history of CML treated with imatinib mesylate presented with lower limb myalgia mainly during the night, inflammatory polyarthralgia of the wrists and metacarpophalangeal joints, and muscle weakness of the thighs. The complaints had begun six months after starting imatinib and worsened over time. There were no relevant comorbidities, tobacco or alcohol consumption. On examination, she had a Manual Muscle Testing-8 (MMT-8) score of 70/80. No other alterations, particularly in the skin, scalp, joints or cardiopulmonary examination were noted.

### 2.2 Diagnostic assessments

**Figure 1:**
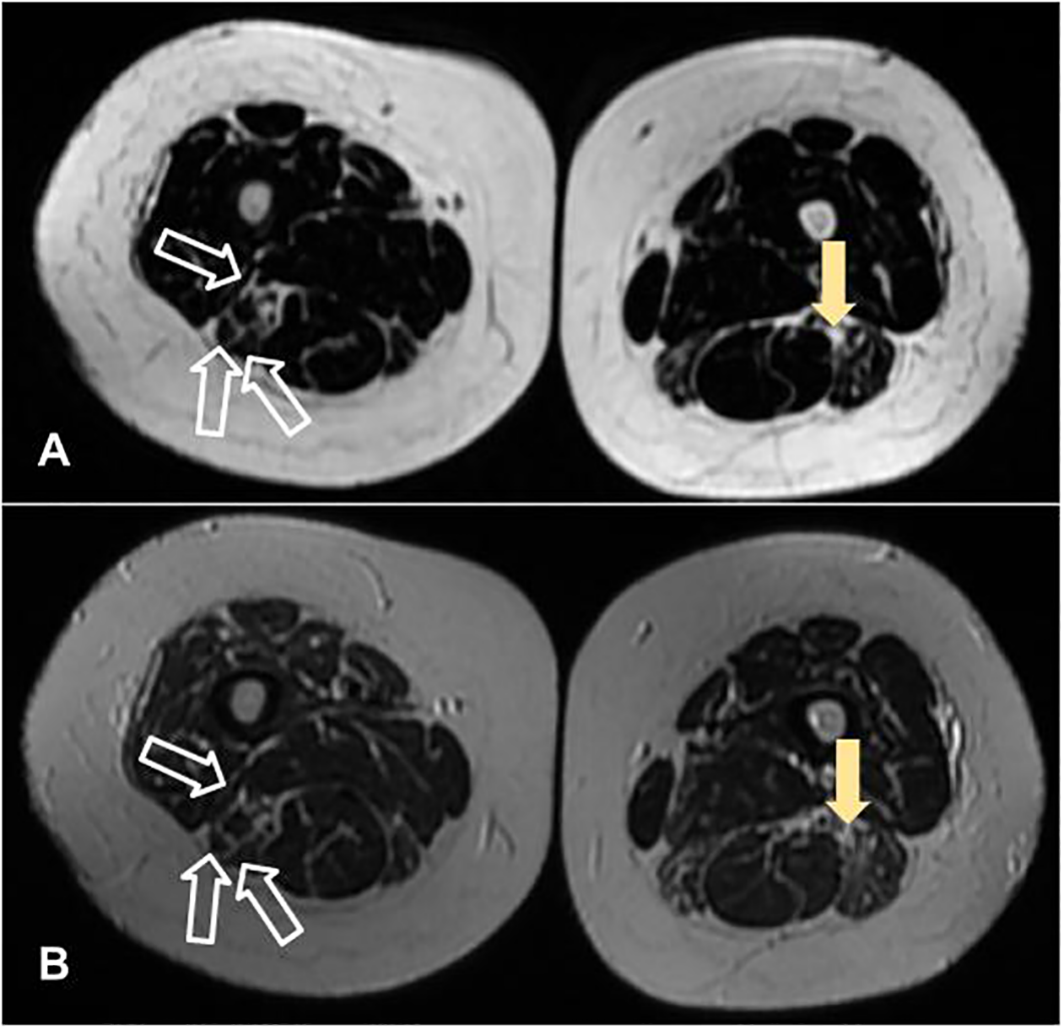
Muscle thigh magnetic resonance imaging scans, in axial plans, revealing fatty infiltration between the fibers (filled arrow) and atrophy of the biceps femoris and semitendinosus muscles (unfilled arrow). **(A, B)** were acquired in LAVA Flex and T2 sequences, respectively.

**Figure 2:**
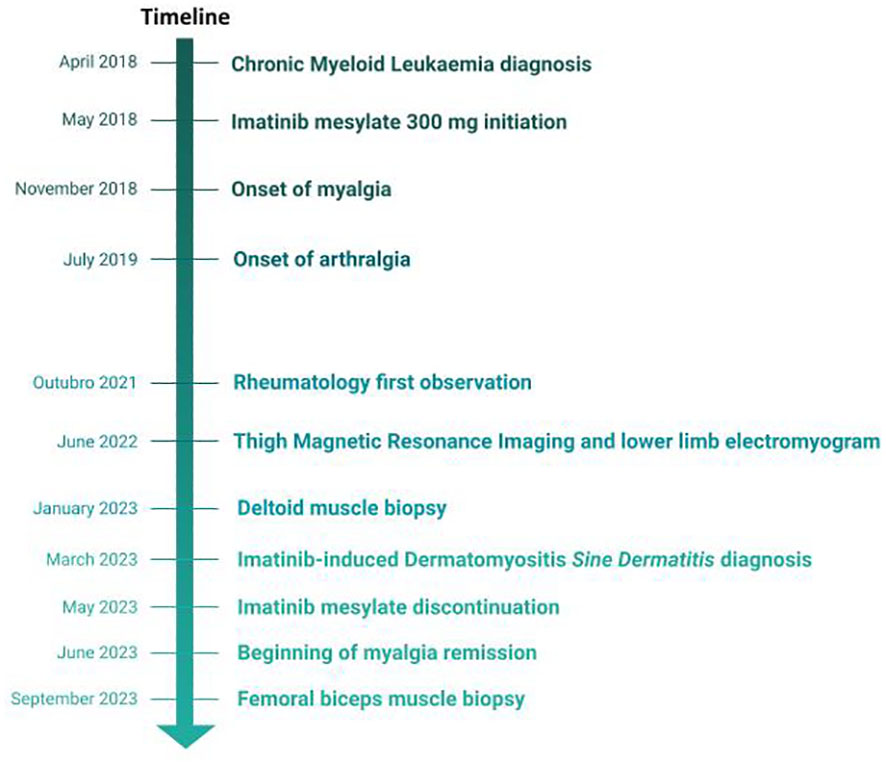
Clinical course of the Imatinib-induced Dermatomyositis *sine dermatitis*.

Blood tests showed anemia and mildly elevated acute phase reactants and muscle enzymes (**Table 1**). Antinuclear antibodies were positive at 1/640 (citoplasmatic linear (AC-15) and nuclear fine speckled (AC-4) pattern), with negative extractable nuclear antigen antibodies, while myopathies panel came strongly positive for anti-Mi2a. Detection electromyography showed rich tracings with a slight increase in polyphasia upon maximum contraction of bilateral vastus medialis, anterior tibialis, trapezii and biceps brachii, without activity at rest, compatible with diffuse muscle fiber damage, without active necrosis. Muscle thigh MRI scans revealed fatty infiltration between the fibers and atrophy of the biceps femoris and semitendinosus muscles (**Figure 1**). According to the Neurology protocol, a deltoid muscle biopsy was performed which revealed a few scattered, non-specific, atrophic fibers suggestive of myositis. **Figure 2** presents the entire clinical course of the reported case.

**Table 1:** Blood test results of the patient.

Parameter	Result	Reference range
Hemoglobin	10.6 g/dL	12-15,3 g/dL
Mean Corpuscular Volume	97.9 fL	80-97 fL
Mean Corpuscular Hemoglobin	33.8 pg	27-33 pg
Leukocytes	5.9x10^9^/L	4-11x10^9^/L
Erythrocyte sedimentation rate	32 mm/h	< 15 mm/h
C-reactive protein	1.56 mg/dL	< 0.5 mg/dL
Creatinine	1.14 mg/dL	0.7-1.2 mg/dL
Aspartate aminotransferase	18 U/L	0-32 U/L
Alanine aminotransferase	11 U/L	0-33 U/L
Lactate dehydrogenase	213 U/L	100-250 U/L
Creatinine kinase	**207 U/L**	26-192 U/L
Aldolase	3.8 U/L	1.2-8.8 U/L
Myoglobin	**68 U/L**	25-58 U/L
Troponin T	**23 ng/L**	< 14 ng/L
Antinuclear antibodies	1/640 (AC-4 and AC-15)	Negative
IIM immunoblot	Strongly positive for anti-Mi2a	Negative

The bold values are those that are high compared to the reference values and are clinically relevant.

### 2.3 Therapeutic interventions and follow-up

Given the mild muscle manifestations, no immunosuppressive or glucocorticoid therapy was started. Following discussion with the assisting hematologist, and considering the sustained clinical and molecular CML remission after five years of treatment, imatinib was discontinued. Approximately four months after stopping imatinib, the patient repeated the biopsy targeting one of the most affected muscles in the MRI scan (which revealed no changes) and showed spontaneous resolution of the myalgia, as well as an improvement in muscle strength (MMT-8 = 74), normalization of muscle enzymes, ANA repetition showed a titre of 1/160 nuclear fine speckled AC-4 pattern and, notably, anti-Mi2a antibodies turned negative. The strict time association between improvement and treatment suspension confirmed the diagnosis of imatinib-induced myositis.

## 3 Discussion

To the best of our knowledge this is the first confirmed report of IIM induced by imatinib described in the literature. The results of EMG, muscle biopsy and MRI scans, combined with the presence of a strongly positive anti-Mi2a autoantibody, strongly supported the diagnosis of IIM. On the other hand, the temporal relationship between the onset of IIM symptoms and imatinib intake, as well as the spontaneous and nearly full recovery of muscle strength and myalgia after its suspension.

Autoantibodies directed against chromodomain helicase DNA binding protein 4 (Anti-Mi2a and Anti-Mi2b) are found in 11–59% of adult DM patients. Clinically, patients with anti-Mi2 can present a range of cutaneous features, but usually have a more favorable prognosis, with mild muscle involvement and a decreased risk of interstitial lung disease and malignancy (9).

Acquired inflammatory myopathies arise from sustained activation of the innate and adaptive immune system, resulting in damage to internal organs. Auto-antibodies are found in a majority of cases, which makes the immune serology an important diagnostic tool (10). Using indirect immunofluorescence, antinuclear antibodies can be detected until 76% of the patients with polymyositis or dermatomyositis, and the most common pattern is a nuclear fluorescence, while the speckled pattern on HEp-2 smears is the most common presentation (11).The International Consensus on ANA Patterns (ICAP) had classified an anti-Mi2-positive pattern into AC-4, also named nuclear fine speckled, which referred to fine tiny speckles throughout the nucleoplasm (12). The patient initially had an ANA titre of 1/640 which, after imatinib suspension, fell to 1/160, maintaining the AC-4 nuclear pattern while the AC-15 cytoplasmic pattern disappeared.

Imatinib mesylate is a selective tyrosine kinase inhibitor targeting the Philadelphia chromosome (BCR-ABL fusion oncoprotein) in CML (7). Despite being well tolerated, myalgias can be frequent and occur in 21% to 52% of patients taking imatinib (8, 13, 14). High CK levels have been reported only rarely (<1%), and rhabdomyolysis was described in a few patients (15, 16). In one anecdotal report, the myositis was possibly coincidental and unrelated to imatinib therapy, since after interrupting imatinib muscle weakness still progressed and serum CKs remained elevated (800-1100 IU/L) (9). Myositis has been reported only rarely in CML, and has been mostly attributed to treatment with hydroxyurea or alpha-interferon.

## 4 Conclusion

We report the first case of DM *sine dermatitis* associated with imatinib. The strong temporal relationship between onset and resolution of signs and symptoms of IIM and the start and discontinuation of imatinib, respectively, strongly suggest a drug-induced etiology. Complementary exams further supported a diagnosis of myositis. In conclusion, this case reveals the importance of a detailed anamnesis and the framing of the temporal and cause-effect relationship in establishing the diagnosis of a drug-associated myositis. This applies even when no similar cases have been described in the literature, underlining the importance of both rheumatologists and hematologists being alert to this type of manifestation.

## Data availability statement

The original contributions presented in the study are included in the article/supplementary material. Further inquiries can be directed to the corresponding author.

## Ethics statement

Written informed consent was obtained from the individual(s) for the publication of any potentially identifiable images or data included in this article.

## Author contributions

AS: Conceptualization, Writing – original draft. VR: Supervision, Validation, Writing – review & editing. RC-M: Supervision, Visualization, Writing – review & editing.
